# Association between Food Store Availability and the Incidence of Functional Disability among Community-Dwelling Older Adults: Results from the Japanese Gerontological Evaluation Cohort Study

**DOI:** 10.3390/nu11102369

**Published:** 2019-10-04

**Authors:** Ryo Momosaki, Hidetaka Wakabayashi, Keisuke Maeda, Hiroshi Shamoto, Shinta Nishioka, Kaori Kojima, Yukako Tani, Norimichi Suzuki, Masamichi Hanazato, Katsunori Kondo

**Affiliations:** 1 Department of Rehabilitation Medicine, Teikyo University School of Medicine University Hospital, Mizonokuchi, Kanagawa 213-8507, Japan; 2 Department of Rehabilitation Medicine, Yokohama City University Medical Center, Kanagawa 240-8555, Japan; noventurenoglory@gmail.com; 3 Department of Palliative and Supportive Medicine, Graduate School of Medicine, Aichi Medical University, Aichi 441-8108, Japan; kskmaeda@aichi-med-u.ac.jp; 4 Department of Neurosurgery, Minamisoma Municipal General Hospital, Fukushima 975-0033, Japan; shahonvs17@gmail.com; 5 Department of Clinical Nutrition and Food Service, Nagasaki Rehabilitation Hospital, Nagasaki 850-0854, Japan; shintacks@yahoo.co.jp; 6 Department of Community Health and Preventive Medicine, Hamamatsu University School of Medicine, Shizuoka 431-3125, Japan; kaworikojima@gmail.com; 7 Department of Global Health Promotion, Tokyo Medical and Dental University, Tokyo 113-8510, Japan; tani.hlth@tmd.ac.jp; 8 Center for Preventive Medical Sciences, Chiba University, Chiba 260-0856, Japan; suzu-nori@chiba-u.jp (N.S.); hanazato@chiba-u.jp (M.H.); kkondo@chiba-u.jp (K.K.); 9 Department of Gerontological Evaluation, Center for Gerontology and Social Science, National Center for Geriatrics and Gerontology, Aichi 474-8511, Japan

**Keywords:** food environment, food stores, functional disability

## Abstract

This study sought to clarify the association between food store availability and the incidence of disability in older adults. This study utilized a population-based cohort study of independent Japanese adults aged ≥65 years, which was a 6 year follow-up of participants in the Japan Gerontological Evaluation Study. A total of 31,273 respondents were extracted. Food store availability was evaluated based on the existence of food stores within 500/1000 m of the home. We utilized participant-reported subjective measurement as well as geographic information system-based objective measurement for the evaluation. The incidence of disability was determined using municipal data on eligibility for long-term care insurance benefits. There were 7643 (24.4%) community-dwelling participants with low subjective food store availability and 5673 (18.1%) with low objective food store availability. During the follow-up period of 6 years, the cumulative incidence of disability was 20.9%, with a significant association between low subjective food store availability and increased disability. Participants who reported low subjective food store availability had a significantly higher likelihood of developing disability (hazard ratio = 1.18, 95% confidence interval: 1.11–1.25) than those who reported high subjective food store availability after adjusting for age, sex, sociodemographic status, environmental status, walking and going out, dietary food intake, body mass index, and comorbidities. Low subjective food store availability was associated with early onset of disability. Accessibility of food stores might contribute to maintaining a disability-free life.

## 1. Introduction

Life expectancy is increasing worldwide, with a resultant increase in the elderly population who are at high risk of physical frailty, sarcopenia, progressive functional disability, dependency, and institutionalization, and will have serious social, medical, and economic impacts [1]. Malnutrition is considered a major risk factor of frailty, sarcopenia, impairment, and disability [2]. The Asia-Pacific Clinical Practice Guidelines for the Management of Frailty provisionally recommend that screening should be performed for frail older adults who have unintended weight loss to identify possible reversible causes and that protein and caloric supplementation/food fortification should be considered in such individuals [3]. The clinical guidelines for sarcopenia state that appropriate nutritional intake could prevent the onset of sarcopenia and is therefore recommended [4]. 

Personal dietary habits, as well as accessibility to food in terms of whether a variety of foods can be obtained, are considered to affect nutritional status. Many older adults face obstacles to accessing food due to a decline in walking and movement ability. As humans, we eat every day, so the food environment can affect us all and has a great influence. People who reside far from a grocery store are reported to have a 25%–46% lower likelihood of healthy dietary habits than those with the most stores nearby among community-dwelling residents in the United States [5]; low subjective accessibility of food increases the risk of low dietary variability among community-dwelling older adults in Japan [6]. According to Sharkey et al., objective and subjective indices of accessibility to a food store, such as further distance to the nearest grocery store, food stores that stock a diverse array of fresh and processed fruit, or those with a wide assortment of fresh and processed vegetables, were associated with reduced regular intake of fruit and vegetables based on data from community residents in the United States [7]. Also, cohort data targeting community-dwelling older adults in Japan showed that living in a food environment located in a poor neighborhood can possibly lead to malnutrition [8], while improved access to food stores was associated with higher body mass index (BMI) [9]. 

Previous studies have shown that the food environment might impact incidence of mortality and dementia in older people [10,11]. If one’s main reason for going out is to shop at a neighborhood grocery store, food store availability might influence the incidence of functional disability in many older adults. However, the relationship between food store availability and functional disability has not been sufficiently verified so far. This study sought to verify the association between food store availability and the incidence of functional disability among older community-dwellers.

## 2. Materials and Methods

### 2.1. Study Population

We used prospective cohort data of a large-scale population-based study that involved older Japanese people aged ≥65 years from the Japan Gerontological Evaluation Study (JAGES) [12].

Data from JAGES were collected from eight municipalities in four prefectures (Hokkaido, Yamanashi, Aichi, and Nagasaki). Baseline data were collected from August 2010 to January 2012. Self-administered questionnaires were sent by mail to independently living individuals in the relevant community. From 52,180 participants who had no certified need for long-term care insurance, we used data on 31,273 participants, after excluding participants with missing information on subjective and/or objective food store availability (20,907 participants). All the participants were duly notified that participation in the JAGES project was voluntary and that returning the completed questionnaire would be considered as consent to participate. Ethics approval (No. 13–14) was obtained from the Ethics Committee at our university. 

### 2.2. Measurements and Variable Definitions

Subjective food store availability was evaluated based on a self-reported questionnaire. The questionnaire consisted of a single question on subjective food store availability: “How many food stores or facilities that sell fresh fruits and vegetables are within 1 kilometer of your house?”, rated on a 4-point scale (“none”, “few”, “some”, “many”) [6]. We defined “many” and “some” as high, and “few” and “none” as low subjective food store availability. 

Objective food store availability was evaluated using geographic information system (GIS)-based methods [13]. Our study utilized 500 m mesh data from the Commercial Establishment Survey of the Ministry of Economy, Trade and Industry [14]. Food stores comprised department stores, general merchandise retailers, convenience stores, and specialty grocery stores. On the premise that all kinds of stores were uniformly distributed in the 500 m mesh, the number of stores along a straight line in a 500 m radius of the residence of each participant was then calculated according to the area of proportional distribution of the map area. All spatial calculations were performed with ArcGIS 10.1 software. Participants who had stores along a straight line within a radius of 500 m from the center of their residential community blocks were categorized as having high objective food store availability and those without stores within a radius of 500 m were categorized as having low objective food store availability. We defined 500 m or less as a reasonable “walkable” distance to a food store, which has been used in similar studies [15,16].

The primary outcome was functional disability, which we defined as certified need for care or support under the long-term care insurance system of Japan [17]. Follow-up of participants was carried out for 6 years based on their incident functional disability. Incident functional disability data was obtained from databases of public municipal long-term care insurance. Participants who had been recently certified as needing care or support through Japan’s long-term care insurance system were deemed as having functional disability [18]. Eligibility for insurance certification is determined based on a national standardized procedure that includes a medical examination by a physician and assessment of physical status and cognitive function.

Demographic variables included age and sex in the baseline survey. Sociodemographic status, walking and going out, driving status, neighborly relationships, nutritional status, and comorbidities were evaluated with a self-reported questionnaire. Variables of social and demographic status were educational attainment, yearly income, living arrangements, employment status, and marital status. Yearly income was adjusted for household size, where income was divided by the square root of the number of individuals per household. Variables of walking as well as going out comprised daily walking time and frequency of going out. For nutritional status, variables were BMI and fruit/vegetable intake and meat/fish intake rate over the previous month. Standard BMI categories were then used to classify participants as obese, overweight, normal, or underweight [19]. Neighborly relationships were evaluated as described by Nakamura et al. [6] by asking about the kind of relationship they have with others in their neighborhood. The responses included the frequency of exchanging greetings or conversing with neighbors and borrowing/lending items, and based on the results, participants were classified as having either a high- or low-level relationship with their neighbors. Variables about comorbidities included therapy for medical conditions/symptoms and symptoms of depression. Multiple answers were permitted, and so participants were required to state whether or not they were at the time receiving medical treatment for malignancy, cardiac disease, stroke, high blood pressure, diabetes mellitus, arthropathy/neuralgia, traumatic fracture, pulmonary disease, and gastrointestinal disorder and dysphagia. Using the Geriatric Depression Scale-15 (Japanese version) [20], symptoms of depression in older adults were assessed and participants were classified into either a non-depressed or depressed group. Cognitive function was categorized as “decline” versus “no decline” and was evaluated with a self-reported questionnaire [21]. Also, we calculated the population density of habitable land in the residential school districts of each participant using the 2010 census results and data for land utilization tertiary mesh (2010) from the National Land Numerical Information of the Ministry of Land, Infrastructure, Transport and Tourism, based on a topographic map of Japan (1:25,000). All non-developed areas, such as rivers, lakes, forests, and wasteland, were excluded from the calculations. All covariates with missing data were classified as “missing.” Categories for each variable are shown in Table 1.

### 2.3. Statistical Analysis

Baseline characteristics of the study participants were defined, and then multivariate cox regression analysis was applied to evaluate the association between food store availability and incident functional disability during the 6 years of follow-up. Four models were generated: Model 1: adjusted for age and sex; Model 2: further adjusted for sociodemographic status (educational attainment, marital status, and employment status, yearly income, and living conditions) as confounding factors to examine whether the relationship between food store availability and disability was independent of other aspects of environmental status; Model 3: additionally adjusted for environmental status (driving status, neighborly relationships, and population density) to investigate whether the relationship between food store availability and functional disability was independent of other environmental factors; and Model 4: further adjusted for walking and going out (daily walking time and rate of going out), dietary food intake (frequency of fruit/vegetable and meat/fish intake), BMI, and comorbidities (malignancy, heart disease, stroke, hypertension, diabetes mellitus, joint disease/neuralgia, traumatic fracture, respiratory disease, digestive disease, dysphagia, depression, and cognitive decline) as potential mediating factors linking food store availability to functional disability. Analyses were performed using SPSS Statistics 19 (IBM Corp., Armonk, NY, USA).

## 3. Results

Table 1 shows the baseline characteristics of the participants and the incidence of functional disability after the 6 year follow-up period. Among all participants (*n* = 31,273, mean age: 74.1 years), 20.5% worked, 84.2% lived with others, 70.1% were married, 7.6% were underweight, 32.5% had cognitive decline, 24.4% had low subjective food store availability, 18.1% had low objective food store availability, and cumulative incidence of functional disability after 6 years was 20.9%. Male, super-old (>80 years) and non-car users tended to have low subjective and objective food store availability. Participants with both low subjective and objective food store availability tended to have low rates of going out. The prevalence of comorbidities was similar between participants with low food store availability and those with high food store availability. Participants with low subjective and objective food store availability tended to have high functional disability.

Results of Cox regression analysis are shown in Table 2. Participants who reported low subjective food store availability had significantly higher likelihood of developing disability (hazard ratio = 1.20, 95% confidence interval: 1.13–1.27; *p* < 0.001) than those who reported high food store availability after age and sex adjustment (Model 1). These hazard ratios were unchanged and remained statistically significant following additional adjustments for sociodemographic and environmental status (Model 2,3). The hazard ratio was slightly reduced but remained statistically significant (hazard ratio = 1.18, 95% confidence interval: 1.11–1.25; *p* < 0.001) after further adjustment for potential mediating factors (walking and going out, nutritional status, and comorbidities) (Model 4). However, objective food store availability showed no significant association with disability after age and sex adjustment in Model 1 (hazard ratio = 0.98, 95% confidence interval: 0.92–1.05; *p* = 0.61). In addition, no significant difference in objective food store availability was evident between Model 2 (hazard ratio = 0.98, 95% confidence interval: 0.92–1.04; *p* = 0.60), Model 3 (hazard ratio = 1.00, 95% confidence interval: 0.93–1.06; *p* = 0.82) and Model 4 (hazard ratio = 1.00, 95% confidence interval: 0.94–1.08; *p* = 0.84).

## 4. Discussion

Low subjective food store availability was associated with early onset of disability among community-dwelling older adults, but this was not the case for objective food store availability. The association remained after adjustments for age, sex, sociodemographic and environmental status, walking and going out, nutritional status, and comorbidities.

As reported by Tani et al., subjectively lower accessibility of healthy food stores measured was associated with mortality [10] and dementia [11], however, they did not examine the association of food store availability and disability. To our knowledge, this is the first report that utilized large-scale data to assess the relationship between food store availability and the incidence of functional disability among older community dwellers.

Earlier studies on food store availability were mainly cross-sectional studies that were carried out in areas of low population density, and by inference in environments with a low food store density (e.g., United States, but the United Kingdom is an exception) [22]. Our study was conducted in Japan within a setting with a substantially high population density and high density of food stores, compared with settings of similar studies in the West. 

Objective and subjective food store availability appear to have different meanings. Food access has five dimensions, comprising availability, accessibility, affordability, accommodation, and acceptability [23,24]. In our study, objective food store availability evaluated only availability of food stores. We found an association between subjective (but not objective) food store availability and onset of disability. This pattern was consistent with findings from previous study, which showed that healthy outcomes were more strongly associated with subjective evaluations of availability but not with objective evaluations [25,26]. Another previous study revealed a high level of mismatch between perceived and objective indices (14%) [25]. Even in areas with low objective food store availability, it is possible for evaluations of subjective food store availability to be high among individuals who have high walking ability, access to public transportation and/or mobile catering options, a person or persons who shops for food on their behalf, or who can grow their own food.

We hypothesized that a food environment that is subjectively evaluated is a better representation of individual differences in self-cultivated food, shopping behaviors, or accepting food from people living close by; not all of these could be addressed by objective evaluation. Several conditions could influence subjective food store availability, for example, traffic around the store, the form of the sidewalk (inclination, stairs), store opening hours/days, security around the store, attributes of other shoppers and store staff, price and assortment of goods, and the store environment (spaciousness of the shop, easy entry, existing amenities such as washrooms and material resources) [27].

The association between subjective food store availability and incidence of functional disability persisted after adjusting for potential mediating factors in Model 4 and could be attributed to other unobserved factors. We speculate that improved food store availability promotes activities of daily living, such as selection of food items, shopping, carrying items, and cooking, which could contribute to maintenance of activities of daily living. Moreover, there is a possibility that we could not subjectively recognize the food stores that sell only low-variety, distasteful, and low-quality foods. Lack of dietary variety has been significantly associated with the progression of frailty in community-dwelling older persons [28]. If food store availability is poor, there is a tendency to stock up on canned foods and ready-to-eat foods and this causes dietary imbalance. Increased opportunities to eat a variety of foods and maintain a well-balanced diet might be responsible for the maintenance of physical status and cognitive function in our study.

Our results suffered from the risk of reverse causation. Participants with many comorbidities and declined physical function might answer low availability of subjective food store and move to a place near the food store. Model 4 included the comorbidities taking the problem into account.

A strength of our study was that we evaluated the impact of subjective, as well as objective, food store availability on disability, investigating these relationships in a high-density setting; using a relatively large sample size added to the statistical power in detecting associations. Nevertheless, there were several limitations in our study. Firstly, the type and size of food stores or the variety, quality, and price of food items were not considered, all of which are possibly significant factors for making healthy food choices. Secondly, the study area may not necessarily be a reflection of Japan as a whole. It is thus necessary for our findings to be reproduced in other regions, particularly in a larger metropolis. Thirdly, home-delivery meals or food services were not taken into account. Fourthly, there is a possibility that food store availability is a product of unmeasured proxy variables related to regional characteristics, such as regional economic affluence and the number of facilities where people gather, which were not adjusted for in the analysis. Fifthly, our database does not contain any time-dependent variables such as the change of food access over 6 years. Sixthly, the self-reported questionnaire that evaluated subjective food store availability did not ask about any other fresh foods than fresh fruits and vegetables. However, we speculated that food stores that sell fresh fruits and vegetables are highly likely to sell other fresh foods.

Low subjective food store availability was associated with early onset of disability, but low objective food store availability was not. Accessibility of food stores might contribute to maintaining a disability-free life. Studies that evaluate the mechanisms involved in the variations in these indices of food store accessibility are required in the future.

## Figures and Tables

**Table 1:** Baseline characteristics of participants and incidence of functional disability after 6 years.

		All Participants	Subjective Food Store Availability		Objective Food Store Availability	
		(*n* = 31,273)	Low (*n* = 7643)	High (*n* = 23,630)	Low (*n* = 5673)	High (*n* = 25,600)
Sex	Male	14,411 (46.1)	3227 (42.2)	11,184 (47.3)	2589 (45.6)	11,822 (46.2)
	Female	16,862 (53.9)	4416 (57.8)	12,446 (52.7)	3084 (54.4)	13,778 (53.8)
Age (years)	65–69	8255 (6.4)	2035 (26.6)	6220 (26.3)	1377 (24.3)	6878 (26.9)
	70–74	9644 (0.8)	2230 (29.2)	7414 (31.4)	1677 (29.6)	7967 (31.1)
	75–79	7390 (23.6)	1794 (23.5)	5596 (23.7)	1406 (24.8)	5984 (23.4)
	≥80	5984 (19.1)	1584 (20.7)	4400 (18.6)	1213 (21.4)	4771 (18.6)
Educational attainment (years)	<6	684 (2.2)	213 (2.8)	471 (2.0)	205 (3.6)	479 (1.9)
	6–9	13,719 (43.9)	3592 (47.0)	10,127 (42.9)	2912 (51.3)	10,807 (42.2)
	10–12	10,606 (33.9)	2447 (32.0)	8159 (34.5)	1622 (28.6)	8984 (35.1)
	≥13	5503 (17.6)	1160 (15.2)	4343 (18.4)	804 (14.2)	4699 (18.4)
	Others/missing	761 (2.4)	231 (3.0)	530 (2.3)	130 (2.3)	655 (2.5)
Employment status	Working	6420 (20.5)	1456 (19.1)	4964 (21.0)	1295 (22.8)	5125 (20.0)
	Retired	17,333 (55.4)	4057 (53.1)	13,276 (56.2)	2710 (47.8)	14,623 (57.1)
	Never worked	3532 (11.3)	1000 (13.1)	2532 (10.7)	708 (12.5)	2824 (11.0)
	missing	3988 (12.8)	1130 (14.8)	2858 (12.1)	960 (16.9)	3028 (11.8)
Yearly income (yen, millions)	<2.00	13,279 (42.5)	3378 (44.2)	9901 (41.9)	2643 (46.6)	10,636 (41.5)
	2.00–3.99	9645 (30.8)	2188 (28.6)	7457 (31.6)	1520 (26.8)	8125 (31.7)
	≥4.00	2732 (8.7)	584 (7.6)	2148 (9.1)	441 (7.8)	2291 (8.9)
	missing	5617 (18.0)	1493 (19.5)	4124 (17.5)	1069 (18.8)	4548 (17.8)
Living conditions	Live alone	4193 (13.4)	1143 (15.1)	3050 (13.0)	712 (12.6)	3481 (13.6)
	Live with others	26,330 (84.2)	6318 (83.2)	20,012 (85.5)	4833 (85.8)	21,497 (84.7)
	missing	487 (1.6)	130 (1.7)	357 (1.5)	90 (1.6)	397 (1.6)
Marital status	Married	21,908 (70.1)	5031 (65.8)	16,877 (71.4)	4006 (70.6)	17,902 (69.9)
	Widowed	6783 (21.7)	1926 (25.2)	4857 (20.6)	1209 (21.3)	5574 (21.8)
	Divorced	1197 (3.8)	314 (4.1)	883 (3.7)	184 (3.2)	1013 (4.0)
	Never married	713 (2.3)	195 (2.6)	518 (2.2)	123 (2.2)	590 (2.3)
	Other/missing	672 (2.1)	177 (2.3)	495 (2.1)	151 (2.6)	521 (2.0)
Driving status	Non-car users	7865 (25.1)	2392 (31.3)	5473 (23.2)	1772 (31.2)	6093 (23.8)
	Car users	10,328 (33.0)	2647 (34.6)	7681 (32.5)	2120 (37.4)	8208 (32.1)
	missing	13,080 (41.8)	2604 (34.1)	10,476 (44.3)	1781 (31.4)	11,299 (44.1)
Neighborly relationships	High level	21,653 (69.2)	5197 (68.0)	16,456 (69.6)	4224 (74.5)	17,429 (68.1)
	Low level	7532 (24.1)	1960 (25.6)	5572 (23.6)	1052 (18.5)	6480 (25.3)
	missing	2088 (6.7)	486 (6.4)	1602 (6.8)	397 (7.0)	1691 (6.6)
Frequency of going out	Everyday	16,031 (51.3)	3320 (43.4)	12,711 (53.8)	2352 (41.5)	13,679 (53.4)
	≥2 times/week	8750 (28.0)	2311 (30.2)	6439 (27.2)	1746 (30.8)	7004 (27.4)
	≤1 time/week	4759 (15.2)	1553 (20.3)	3206 (13.6)	1265 (22.3)	3494 (13.6)
	missing	1733 (5.5)	459 (6.0)	1274 (5.4)	310 (5.5)	1423 (5.6)
Daily walking time	≥1 h/day	9139 (29.2)	2127 (27.8)	7012 (29.7)	1731 (30.5)	7408 (28.9)
	<1 h/day	20,249 (64.7)	5007 (65.5)	15,242 (64.5)	3577 (63.1)	16,672 (65.1)
	Missing	1885 (6.0)	509 (6.7)	1376 (5.8)	365 (6.4)	1520 (5.9)
Frequency of meat/fish intake	≥1 time/day	12,126 (38.8)	2658 (34.8)	9467 (40.1)	2198 (38.7)	9928 (38.8)
	<1 time/day	17,273 (55.2)	4469 (58.5)	12,804 (54.2)	3148 (55.5)	14,125 (55.2)
	missing	1874 (6.0)	516 (6.8)	1358 (5.7)	327 (5.8)	1547 (6.0)
Frequency of vegetable/fruit intake	≥1 time/day	23,508 (75.2)	5495 (71.9)	18,013 (76.2)	42.7 (74.2)	1931 (75.4)
	<1 time/day	6075 (19.4)	1697 (22.2)	4378 (18.5)	1175 (20.7)	4900 (19.1)
	missing	1690 (5.4)	451 (5.9)	1239 (5.2)	291 (5.1)	1399 (5.5)
Body mass index	Underweight < 18.5	2367 (7.6)	589 (7.7)	1778 (7.5)	410 (7.2)	1957 (7.6)
	Normal 18.5–24.9	21,433 (68.5)	5228 (68.4)	16,205 (68.6)	3750 (66.1)	17,683 (69.1)
	Overweight 25–29.9	5767 (18.4)	1367 (17.9)	4400 (18.6)	1157 (20.4)	4610 (18.0)
	Obese ≥ 30	732 (2.3)	203 (2.7)	529 (2.2)	141 (2.5)	591 (2.3)
	missing	974 (3.1)	256 (3.3)	718 (3.0)	215 (3.8)	759 (3.0)
Comorbidities	Malignancy	1383 (4.4)	351 (4.6)	1032 (4.4)	229 (4.0)	1154 (4.5)
	Heart disease	3785 (12.1)	972 (12.7)	2813 (11.9)	677 (11.9)	3108 (12.1)
	Stroke	4.7 (1.3)	88 (1.2)	319 (1.3)	76 (1.3)	331 (1.3)
	Hypertension	12,330 (39.4)	3107 (40.7)	9223 (39.0)	2302 (40.6)	10,028 (39.2)
	Diabetes mellitus	2819 (12.2)	896 (11.7)	2923 (12.4)	644 (11.4)	3175 (12.4)
	Joint disease/ Neuralgia	1242 (4.0)	369 (4.8)	873 (3.7)	242 (4.3)	1000 (3.9)
	Traumatic fracture	2024 (6.5)	567 (7.4)	1457 (6.2)	4.3 (7.1)	1621 (6.3)
	Respiratory disease	652 (2.1)	163 (2.1)	489 (2.1)	123 (2.2)	529 (2.1)
	Digestive disease	307 (1.0)	87 (1.1)	220 (0.9)	50 (0.9)	257 (1.0)
	Dysphagia	191 (0.6)	74 (1.0)	117 (0.5)	34 (0.6)	157 (0.6)
Depression	Non-depressed	18,831 (60.2)	4056 (53.1)	14,775 (62.5)	3381 (59.6)	15,450 (60.4)
	Depressed	7285 (23.3)	2247 (29.4)	5038 (21.3)	1364 (24.0)	5921 (23.1)
	missing	5157 (16.5)	1340(17.5)	3817 (16.2)	928 (16.4)	4229 (16.5)
Cognitive function	Decline	10,158 (32.5)	2673 (35.0)	7485 (31.7)	1902 (33.5)	8256 (32.3)
	Not decline	21,115 (67.5)	4970 (65.0)	16,145 (68.3)	3771 (66.5)	17,344 (67.8)
Incidence of functional disability	Non-certification	24,741 (79.1)	5800 (75.9)	18,941 (80.2)	4451 (78.5)	20,290 (79.3)
	Certification	6532 (20.9)	1843 (24.1)	4689 (19.8)	1222 (21.5)	5310 (20.7)

Categorical data are expressed as a number (%).

**Table 2:** Hazard ratios with 95% confidence intervals for incident functional disability with objective and subjective food store availability.

		Model 1 HR (95% CI)	Model 2 HR (95% CI)	Model 3 HR (95% CI)	Model 4 HR (95% CI)
Subjective food store availability	High	Reference	Reference	Reference	Reference
	Low	1.20 (1.13 to 1.27)	1.20 (1.13 to 1.26)	1.20 (1.13 to 1.26)	1.18 (1.11 to 1.25)
Objective food store availability	High	Reference	Reference	Reference	Reference
	Low	0.98 (0.92 to 1.05)	0.98 (0.92 to 1.04)	1.00 (0.93 to 1.06)	1.00 (0.94 to 1.08)

HR hazard ratio, CI confidence interval, ref reference group. Model 1: Adjusted for age and sex. Model 2: Model 1 + adjustments for sociodemographic status (education attainment, marital status, employment status, yearly income, living situation). Model 3: Model 2 + adjustments for environmental status (driving status, neighborly relationships, and population density). Model 4: Model 3 + adjustments for walking and going out (daily walking time and rate of going out), dietary food intake (frequency of fruit/vegetable and meat/fish intake), body mass index, and comorbidities (malignancy, heart disease, stroke, hypertension, diabetes mellitus, joint disease/neuralgia, traumatic fracture, respiratory disease, gastrointestinal disease, dysphagia, depression, and cognitive decline).
